# Is there any effect of hcc location on selective internal radiation therapy with ^90^yttrium response?

**DOI:** 10.1007/s12149-025-02028-5

**Published:** 2025-02-27

**Authors:** Elife Akgun, Cagrı Erdim, Burcu Ibicioglu, Tevfik Guzelbey, Burcu Esen Akkas, Ozgur Kılıckesmez

**Affiliations:** 1 Department of Nuclear Medicine, University of Health Sciences Türkiye, Basaksehir Cam and Sakura City Hospital, Istanbul, Türkiye; 2 Department of Radiology Division of Interventional Radiology, University of Health Sciences Türkiye, Basaksehir Cam and Sakura City Hospital, Istanbul, Türkiye

**Keywords:** Hepatocellular carcinoma, Yttrium, Radioembolization, Location, Central, Peripheral

## Abstract

**Background:**

We planned this retrospective study to evaluate the effect of the central vs peripheral location effect on the success of selective internal radiation therapy (SIRT) with ^90^Yttrium-90 (^90^Y) glass microspheres in hepatocellular carcinomas (HCC).

**Material and methods:**

Thirty-eight patients diagnosed with HCC who were eligible for SIRT with ^90^Y glass microspheres were included in this study. The location being central versus peripheral was defined as explained: Straight lines through the bifurcation of the right and left branches of the portal vein to the center of the HCC and the peripheral surface of the liver were traced on the same plane. The coefficient was determined as a ratio of the center of the HCC to the distance from the hilum of the liver at the portal vein bifurcation. Value under ½ accepted as central location (Group 1, *n* = 17), over ½ values are accepted as peripheral location (Group 2, *n* = 21). Treatment responses were analyzed after 2 months of the treatment with magnetic resonance imaging, and 2-deoxy-2-[^18^F]fluoro-D-glucose positron emission tomography/computed tomography ([^18^F]FDG PET/CT) for FDG-avid ones. Differences in treatment responses rates, treatment approach, the absorbed doses and the volumes of each liver segments between groups were investigated.

**Results:**

In Group 1; mean age was 67. In 5 cases split infusion, in 10 cases tumor selective treatment approach were applied. According to PERCIST/mRECIST criteria treatment responses categories: complete response in 2/1 cases, partial response in 7/9 cases, stable disease in 3/4 cases, progressive diseases in 2/3 cases; respectively. AFP value decreased in 2 cases, increased in 7 cases, and was stable in 1 case. Mean absorbed doses were 347.9 Gy for tumor, 140.6 Gy for perfused normal tissue, and 26.1 Gy for the normal liver. In Group 2; the mean age was 71.5. In 5 cases split infusion, and in 1 case non-selective treatment approach were applied. According to PERCIST/mRECIST criteria treatment responses categories: complete response in 7/6 cases, partial response in 7/10 cases, stable disease in 2/2 cases, and progressive diseases in 3/3 cases; respectively. AFP value decreased in 9 cases, increased in 2 cases, and was stable in 2 cases. Mean absorbed doses were 495.9 Gy for tumor, 134 Gy for perfused normal tissue, and 17.3 Gy for the normal liver.There is no statistically significant difference in terms of gender, treatment response rates, tumor volumes, perfuse tissue volumes between 2 groups. However, tumor-selective approach and absorbed doses of the perfused normal tissue and the tumor were significantly higher in Group 2 (*p* = 0.007, 0.04, and 0.02; respectively).

**Conclusion:**

Contrary to expectation, centrally located HCCs could be treated as successfully as peripherally located HCCs. However, the complete response rate in the peripheral located tumor is more frequent than centrally located ones.

## Introduction

Hepatocellular carcinoma (HCC) is the most common type of primary liver cancer, accounting for 75%80% of all liver cancer. Its onset generally linked with viral hepatitis, and alcoholic fatty liver (1). Mortality rate related to this malignancy is quite high. For early-stage disease surgical resection is the primary treatment modality. However, a significant proportion of patients presented with intermediate or advanced-stage disease, thereby rendering them amenable to surgical resection (2). Despite new targeted treatment options, it is still one of the leading causes of cancer-related death worldwide.

Although the portal vein is the primary blood source of the normal liver parenchyma, liver tumors bigger than 2 cm in sized are supplied by the hepatic artery. This unique vascularity makes possible delivering radioactive microspheres intraarterially. ^9o^Yttrium (^90^Y) is the most used radionuclide for selective internal radiation therapy (SIRT). The main purpose of dosimetry is planning reasonably higher tumor-absorbed dose while sparing normal parenchyma. Especially for centrally located tumors which are supplied by the proximal proportion of arteries, it is sometimes not possible. Transient embolization of normal tissue vascular structure could be a safe option for saving normal tissue. However, it requires an experienced interventional radiologist. In some of these cases, in order not to increase perfuse normal tissue absorbed dose and not to cause radiation-related hepatic injury, the tumor absorbed dose may have to be reduced. As well known, low tumor dose is reduced objective radiation treatment responses.

In this retrospective study, we aim to investigate the effect of HCC localization on SIRT with ^90^Y glass microsphere response in HCCs.

## Material and methods

### Study population

Patients diagnosed with HCC based on histopathologic examination or serum alfa-fetoprotein level (AFP) and hepatocyte-specific contrast agent enhanced magnetic resonance images (MRI) were included in this study. None of these patients revealed any local or systemic treatment before. All patients who were not candidates for surgery due to the disease stage or other comorbities were considered as a candidate for SIRT with ^90^Y glass microsphere in a multidisciplinary tumor board. Hepatic reserve was at least 30% of the whole liver.

All serum laboratory test results were recorded. Adequate bone marrow function and renal function were verified. Albumin-bilirubin (ALBI) scores were calculated using this formula the formula—ALBI score = (log10 bilirubin μmol/L × 0.66) + (− 0.085 × albumin g/L). The score is then graded according to the calculated value—ALBI grade 1: score ≤ −2.60; ALBI grade 2:− 2.60 < score ≤  −1.39; ALBI grade 3: score > −1.39.

A total of 38 cases diagnosed with HCC and treated with SIRT with ^90^Y glass microsphere were included in the study. The median age was 64.5 year-old (range: 14–85; 27 males, 11 female). Median targeted tumor volume was 108 cc (range: 14–1700 cc). All patients Child Pugh scores were under B7, ALBI grades were under 2. None of the cases suffered from alcoholic liver disease. Fifteen of all cases were diagnosed with hepatitis (HBV:11, HCV:4).

To evaluate the aggressiveness of HCC and evaluate extrahepatic metastasis, 2-deoxy-2-[^18^F]fluoro-D-glucose positron emission tomography/computed tomography ([^18^F]FDG PET/CT) was performed without contrast agents (Philips Medical Systems, OH, USA).

MRI performed before and 8 weeks after the treatment. None of cases have portal vein thrombosis. Treatment responses were evaluated on a per nodule basis, according to the Modified Response Evaluation Criteria in Solid Tumors (mRECIST) (3). Cases with FDG-avid lesions underwent ([^18^F]FDG PET/CT 8 weeks after the treatment. FDG-avid lesions treatment responses were classified according to Positron Emission Tomography Response Criteria in Solid Tumors (PERCIST) (4). The lowest AFP value was recorded in the 8-week period after treatment. Follow-up AFP values scoring according to: 1: Initial value is low, 2: Decreasing, 3: Increasing, 4: Stabile.

### ^***90***^***Y Glass radioembolization procedure***

Experienced interventional radiologists performed hepatic artery angiography. Feeding artery of the target lesions were selectively catheterized as much as possible. Selective-nonselective, split treatment options were chosen according to angiography findings, and vascular accessibilities. 185 MBq ^99m^Technetium macro-aggregated albumin ([^99m^Tc]Tc-MAA) was injected through each feeding arteries. Before each injection cone beam CT performed. After bleeding control, scintigraphies were revealed to assess tumor perfusion, extrahepatic radionuclide leakage, lung-liver shunt fraction. Cases in which increased activity uptake was observed in less than 80% of the tumor volume or in which activity uptake was detected at a higher degree in perfused normal tissue than the tumor were considered not eligible for the treatment.

^90^Y radioembolization treatment was planned using with Simplicit90Y™ program (Mirada Medical LTD., Oxford, UK) according to the volumetric multi-compartmental dosimetric approach. MRI or contrast-enhanced CT images were used for the segmentation of the liver, the tumor, and the normal liver. The perfused liver volume, tumor volume, perfused normal liver volume (perfused but nontumoral liver) and tumor coverage on the MAA perfusion scan was controlled. Absorbed doses for each liver segments, the perfused liver, the whole liver, the tumor (TAD), the perfused normal liver (PNAD), the normal whole liver (whole liver without tumor) (NLAD), and lung, were calculated by two experienced nuclear medicine specialists and a medical physicist. The patient was deemed eligible for ^90^Y microsphere therapy if the predicted lung absorbed dose < 30 Gy, tumor volume coverage by MAA activity is optimal and no activity deposition was observed on extra-hepatic area.

By same interventional radiologist who performed the first angiography, using the calculated activity at the same catheter position as the [^99m^Tc]Tc-MAA study, ^90^Y glass microsphere (TheraSphere™, Boston Scientific Corp., Marlborough, MA) was infused approximately two weeks after the shunt study.

The day after the treatment, PET/CT was performed to assess microsphere distribution and any potential extrahepatic activity (5). Posttreatment images were acquired on Philips Ingenuity TF −64 PET/CT scanner (Philips Medical Systems, OH, USA) using 20 min per bed position (288 × 288 pixels, 2 × 2 mm) with a slice thickness of 2 mm.

### Classification of lesion localization

The location of the lesions was determined using the dynamic hepatocyte specific contrast-enhanced MRI. The location being central versus peripheral was determined on coronal images Contrast enhanced T1 weighted.

**Fig. 1:**
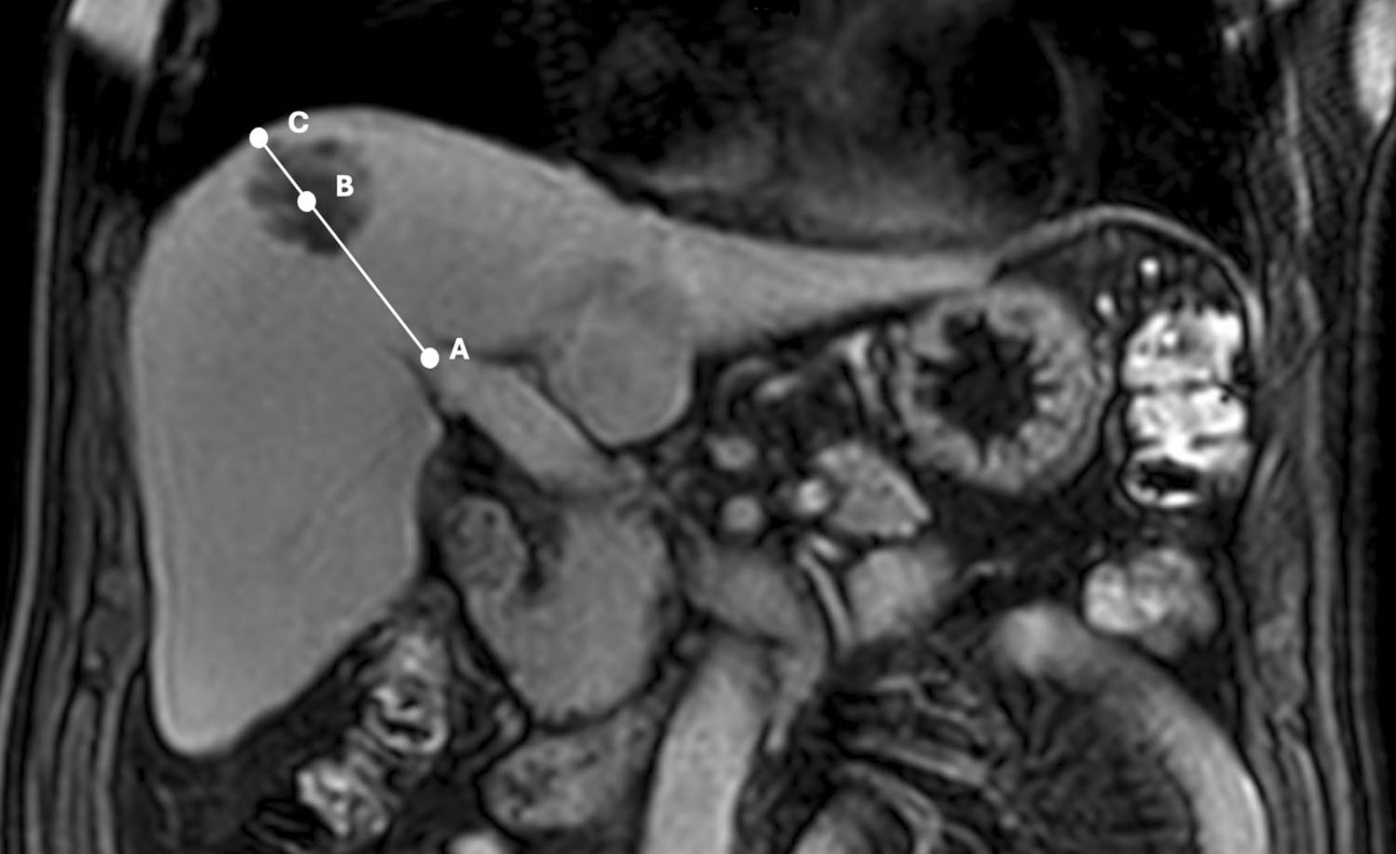
A late venous phase contrast-enhanced coronal T1 weigheted MR image. HCC location coefficient = distance from the bifurcation of the portal vein divided by the diameter of the whole liver on the same line (AB/AC). *MR* Magnetic resonance

Center of the HCC defined as the intersection of the long and short axis’s in the maximum cross-section of the lesion. Straight lines through the bifurcation of the right and left branches of the portal vein to the center of the HCC and the peripheral surface of the liver were traced on the same plane (Fig. 1). The coefficient was determined as a ratio of the center of the HCC to the distance from the hilum of the liver at the portal vein bifurcation. Value under ½ accepted as central location (Group 1; *n* = 17), over ½ values are accepted as peripheral location (Group 2; *n* = 21).

This retrospective study was conducted with the approval of our institutional review board. Due to retrospective nature, the requirement for informed consent was waived.

All procedures performed in studies involving human participants were in accordance with the ethical standards of the institutional and/or national research committee and with the 1964 Helsinki Declaration and its later amendments or comparable ethical standards.

### Statistical analysis

Patients were grouped into central/peripheral location, right/left lobe location, selective/non-selective approach, single session/split infusion patterns. It was investigated whether there were differences between these groups in terms of demographic datas, absorbed doses and volumes of tumor, perfused normal liver, normal whole liver, treatment responses, scores of AFP values.

The Mann–Whitney U test, and Kruskal–Wallis test were used to assess the association between.

response to SIRT with ^90^Y glass microsphere and different variables. Correlations were investigated using with Spearman correlation test. All analyses were performed using SPSS software version 26.0. The results of the statistical analyses were considered significant at a p value < 0.05.

## Results

According to mRECIST criteria, in 7 HCC lesions complete response was achieved 8-weeks after treatment. While 19 lesions showed partial response to treatment, 6 lesions were evaluated as stable, and 6 lesions were evaluated as progressive. Table 1 summarizes the demographic data, treatment approaches, the absorbed doses and the volumes of each liver segments, and treatment responses of both groups.

**Table 1:** Patient demographics and clinical characteristics

Central location group 1 *n* = 17		Periferal location group 2 *n* = 21	*p*-value
Age			
*mean* ± *SD*	67 ± 17.8	71.5 ± 5.7	0.38
Gender	5 F/12 M	6 F/15 M	0.95
Right Lobe/Left Lobe *n*	Right Lobe: *n* = 11 Left Lobe: *n* = 6	Right Lobe: *n* = 16 Left Lobe: *n* = 5	0.37
Single/Split enjection *n*	Single: *n* = 12 Split:*n* = 5	Single: *n* = 16 Split:*n* = 5	0.70
Selective/Non-selective approach *n*	Selective: *n* = 10 Non-selective: *n* = 7	Selective: *n* = 20 Non-selective: *n* = 1	0.007*
Perfused normal liver absorbed dose			
*mean* ± *SD (Gy)*	140.6 ± 68.9	208.0 ± 130.5	0.04*
Perfused normal liver volume			
*mean* ± *SD (cc)*	245.8 ± 301.7	134.0 ± 191.0	0.34
Tumor absorbed dose			
*mean* ± *SD (Gy)*	347.9 ± 133.8	495.8 ± 180.7	0.02*
Tumor volume			
*mean* ± *SD (cc)*	321.8 ± 430.9	113.1 ± 90.6	0.22
Normal whole liver absorbed dose			
*mean* ± *SD (Gy)*	26.1 ± 24.3	17.3 ± 17.0	0.38
Lung absorbed dose			
*mean* ± *SD (Gy)*	8.5 ± 11.3	3.7 ± 4.4	0.46
mRECIST			
1: Complete response	1: *n* = 1	1: *n* = 6	0.06
2: Partial response	2: *n* = 9	2: *n* = 10	
3: Stabile disease	3: *n* = 4	3: *n* = 2	
4: Progressive disease	4: *n* = 3	4: *n* = 3	
PERCIST			
1: Complete response	1: *n* = 2	1: *n* = 7	0.50
2: Partial response	2: *n* = 7	2: *n* = 7	
3: Stabile disease	3: *n* = 3	3: *n* = 2	
4: Progressive disease	4: *n* = 2	4: *n* = 3	
5: Non-FDG avid	5: *n* = 3	5: *n* = 2	
AFP response			
1: Initial value is low	1: *n* = 7	1: *n* = 8	0.51
2: Decreasing	2: *n* = 2	2: *n* = 9	
3: Increasing	3: *n* = 7	3: *n* = 2	
4: Stabile	4: *n* = 1	4: *n* = 2	

*Gy* Gray, *cc* Cubic centimetre, *AFP* Alfa-fetoprotein, *mRECIST*: Modified response evaluation criteria in solid tumors, *PERCIST* Positron emission tomography response criteria in solid tumors

^*^ Statiscially significant

In Group 2 selective treatment approach rate was significantly higher compared with Group 1 (*p* = 0.007). Related to this result; PNAD, and TAD were higher in Group 2 (*p* = 0.045, 0.02; respectively). Consequence of higher TAD, number of the cases with complete response to treatment was higher in Group 2 (Group 1 *n* = 1, Group 2 *n* = 6). However, response to the treatment did not change significantly between the groups.

When patients grouped as right/left lobe location (mean age 66.5/61.4; male *n* = 18/9; TAD 419.4/454.5 Gy; PNAD 191.5/149.1 Gy; complete response *n* = 4/3; partial response *n* = 16/3; stabile disease *n* = 2/4; progressive disease *n* = 4/2; for right/left lobe respectively), single/split infusion (mean age 65.9/62.7; male *n* = 22/5; TAD 424.9/442.9 Gy; PNAD 164.5/217.6 Gy; complete response *n* = 5/2; partial response n = 15/4; stabile disease *n* = 4/2; progressive disease *n* = 4/2; for single/split infusion respectively); demographic data’s, liver radiation absorbed doses, treatment responses did not differ significantly.

**Fig. 2:**
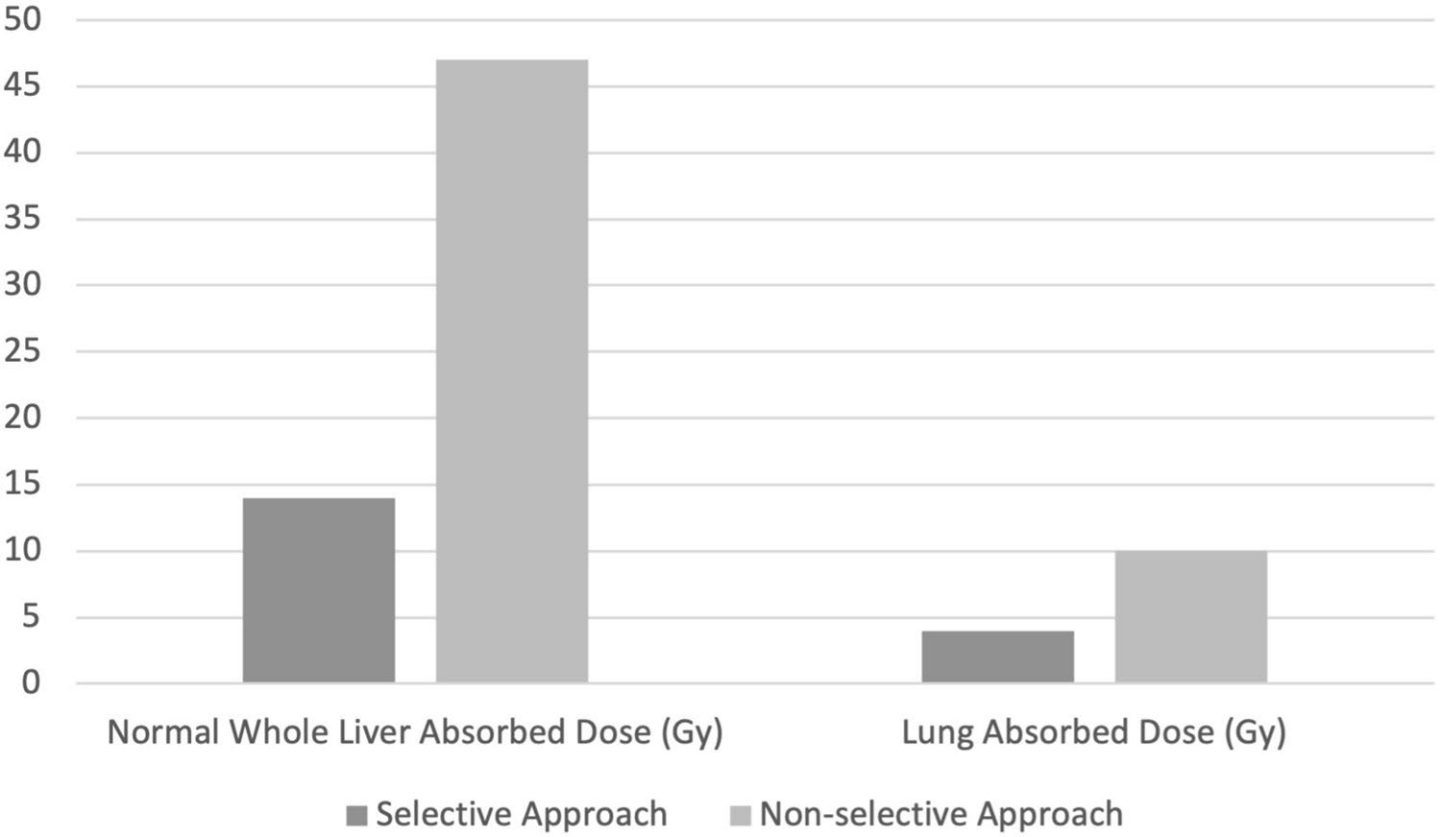
Bar chart showing the absorbed of some liver segments in selective/nonselective approach groups. *Gy* Gray

**Fig. 3:**
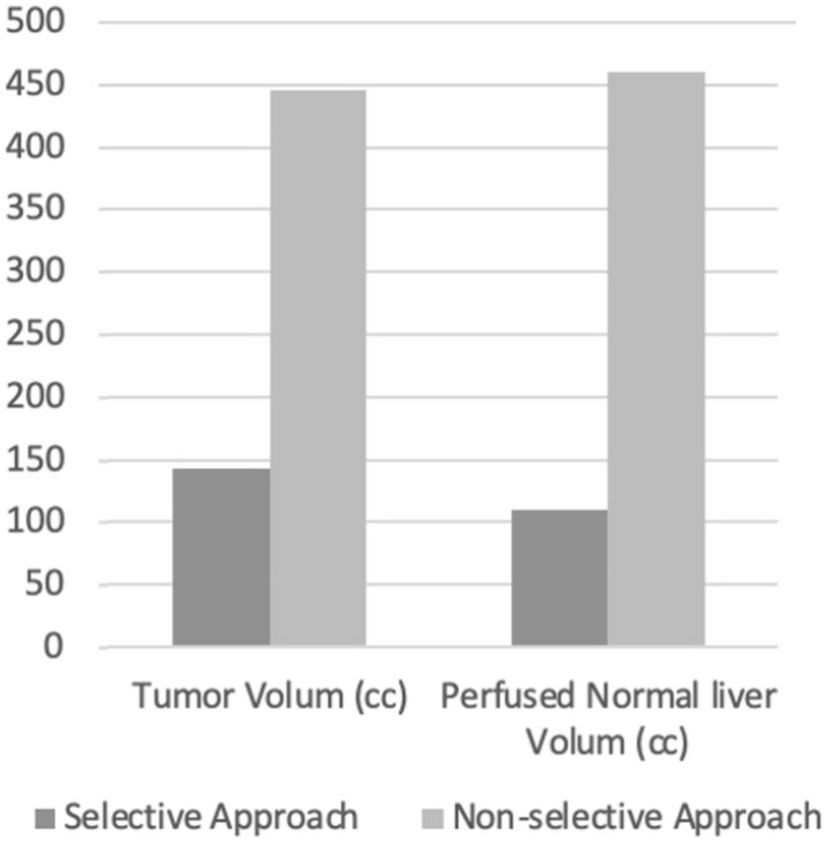
Bar chart showing the volumes of some liver segments in selective/nonselective approach groups. *cc* CUBİC centimetre

The tumor volume, perfused liver volume, perfused normal liver volume; NLAD, and lung absorbed dose were significantly higher in cases treated with a non-selective approach (142.3/445.9 cc, *p* = 0.002; 252.8/906.3 cc, *p* < 0.001; 110.2/460.8 cc, *p* = 0.01; 14/46.9 Gy, *p* = 0.001; 4.2/10.1 Gy *p* = 0.04; for selective/non-selective approach respectively) (Fig. 2 and 3). There was no statistically significant difference between the selective/non-selective approaches groups regarding single/split infusion (22/8 for selective approach, 6/2 for non-selective approach), PNAD (190.1/130.7 Gy), TAD (453.2/341 Gy), radiologic (complete response n = 6/1; partial response n = 15/4; stabile disease *n* = 3/3; progressive disease *n* = 6/0; for selective/non-selective approach respectively) and laboratory treatment responses (decreasing 9/2, increasing 5/4, stabile 3/0; for selective/non-selective approach respectively).

Complete and partial treatment response rates according to mRECIST criteria were significantly higher in cases with decreasing AFP value comparing with cases with stable and increasing AFP values (p = 0.008). While PERCIST scores did not correlate with AFP values significantly, mRECIST scores and AFP values were positively moderately correlated (rho:0.34, *p* = 0.04).

**Fig. 4:**
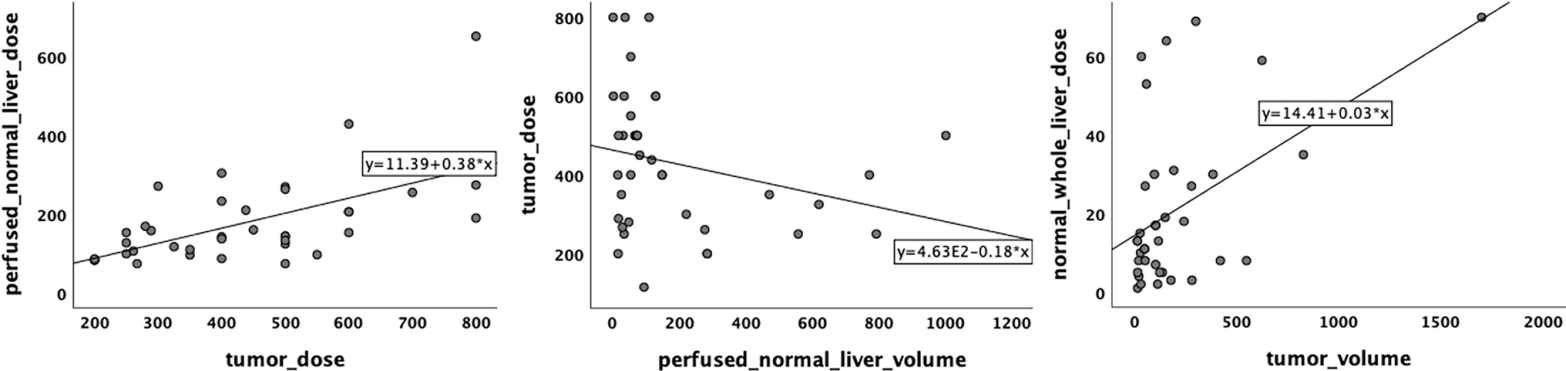
Scatter plot graphics demonstrating correlation between absorbed doses and volumes of some liver segments

Between TAD and PNAD moderately significant positive correlation was detected (rho:0.56, *p* =  < 0.001). The increase in the volume of perfused liver resulted in a reduction of the absorbed dose of the tumor (rho: −0.40, *p* = 0.01). NLAD was positively correlated with the tumor volume, the perfused liver volume, and the perfused normal liver volume (rho: 0.38, *p* = 0.02; rho: 0.79, *p* =  < 0.001; rho: 0.89, *p* =  < 0.001). The perfused liver volume was positively correlated with both the volume of the tumor and perfused normal liver (rho: 0.69, *p* =  < 0.001; rho: 0.74, *p* =  < 0.001) (Fig. 4).

## Discussion

Due to unique vascular physiology, SIRT with ^90^Y microsphere is a popular treatment option in hypervascular liver malignities like HCCs. Although many clinics, laboratories, histopathologic and radiologic features were analyzed regarding their effect on SIRT response, the effect of the central vs peripheral location was not searched enough (6).

In our cohort, we include similar size of tumor in each central and peripheral located groups. Because tumor size is an important factor in angiographic procedure and radiation dose calculation.

Peripheral zones of the liver had less vascularity compared to the central zones. ^90^Y is a catheter based brachytherapy with minimal embolic effect. Free oxygen radicals are the main responsible for radiation-induced damage. It means less vascularity produce a worse treatment response. In our series, there was no statistically significant difference between the central and the peripheral location groups regarding the treatment response rates. However, complete response rates were significantly higher in peripheral-located tumors. Considering the radiation biologic effect, this result could be considered as contradictory to the knowledge about the less vascularity in the periphery. Herein, we think that the main factor affecting the treatment response is the TAD which is significantly higher in the peripheral-located tumors compared with the central-located ones (495.8 Gy vs 347.9 Gy). It is well known that tumor-absorbed doses significantly improve the objective response rate in HCCs (7). As we know that 400 Gy is the threshold dose for an ablative effect (8), it is an expected finding that the complete treatment response rate be higher in the peripheral located cases whose tumor absorbed dose were above 400 Gy. However, we evaluate treatment response 2 months after treatment which is actually a very short time for all radiation treatments. Maybe some cases with partial treatment response will be categorized into complete responder group at later times.

The aim of the SIRT is to deliver tumoricidal radiation dose to tumor while sparing the non-tumoral liver. Patients with HCC generally have underlying liver parenchymal disease which makes every volume of the normal liver tissue valuable. In order not to decompensate already existing chronic liver disease, ALBI scores which reflect liver function deuteration earlier than Child Pugh scores should be closely monitor; and also absorbed dose of the non-tumoral liver tissue should be calculate according to clinic-laboratory results, liver segment volumes. The central located tumors generally feed with branch of the left and right hepatic artery which sometimes makes angiographic procedure more complicated, causes increase in the volume of the perfused normal liver. In our series, between the groups single-split infusion rates did not differ significantly. We planned SIRT with ^90^Y with ablative intention in this study. To decrease perfused normal liver volumes and to increase TAD, our interventional radiology team generally selectively catheterized of the tumor arteries even in peripheral located tumors. That’s why split infusion rates were similar between the groups.

The similarity of split/single infusion rates in selective and nonselective approach groups suggests that the type of infusion choice is made according to tumor cover status, not the approach.

Although volumes of the liver segments were significantly higher in the nonselective approach group, TAD and response rates did not differ significantly between the selective-nonselective approach group. We think it is a benefit of personalized multi-compartmental volumetric dosimetric approach. In the past, ampric dose calculation resulted in low TAD which has probably no tumoricidal effect (9). With increasing knowledge about dosimetry, we are aware that absorbed doses of the tumor and normal liver segments should be balanced.

Even if it is not to a significant degree, the volume of the perfused normal liver was less in peripheral located tumors. Differences in the PNADs between the groups were significant which was higher in peripheral-located tumors. In some cases to increase TAD, and the success of the SIRT higher PNAD could be planned. None of our cases suffer from radiation induced liver disease.

Even though several studies investigated the upper limit of dose to the non-tumoral liver, this issue continues to be unclarified. Chiesa et al. suggested that non-tumoral liver-absorbed doses should be planned according to baseline bilirubin levels in patients treated with ^90^Y glass microspheres by lobar injection. (10). They reported that less than 50 Gy for patients with baseline bilirubin higher than 1.1 mg/dL was safe. They recommended not to exceed 90 Gy in patients with baseline bilirubin lower than 1.1 mg/dL. In our cohort, NLADs were well under these limits.

Although non-selective approach rate was higher in centrally located group perfused normal liver volume did not differ significantly between each tumor. It means with the split infusion option; we could reduce perfused nontumoral liver volume.

Elsahhar et al. demonstrated that the peripheral or central location of HCC has no effect on the success of transarterial chemoembolization (TACE) treatment (11). Contrary to this result, Miki et al. reported that peripheral-located tumors complete response rates after TACE were higher compared with central ones (12). According to us, comparing these studies with ours is not a correct approach. Because the acting mechanisms of TACE and SIRT are completely opposite to each other. Particles size used in TACE is huge which is responsible embolic effect. Contrary to TACE, SIRT with ^90^Y microsphere has a minimal embolic effect. Actually, embolization reduces effectiveness of the radiation therapy by reducing the amount of oxygen.

We think that the results of this study should not generalize to metastatic lesions especially the hipovascular ones. In HCCs, which is in the hypervascular tumor group, a large portion of the given activity is localized into the tumor rather than the normal liver. Due to this high tumor/normal perfused tissue ratio, it is possible to reach high tumoral absorbed doses sparing normal liver tissue with the multicompartment volumetric dosimetric approach even in non-selective treatment approaches in central located tumors.

There are some limitations of this study. First, our study was limited by its retrospective nature as well as the relatively small size of the cohort. Results of this study should be confirmed with a larger cohort. Second, the follow-up period was relatively short for radiation therapies. Some of partial responder may be reveal complete treatment response during the follow-up. Finally, etiology of the cirrhosis was not included in this study because of the small number of population.

## Conclusion

SIRT with ^90^Y microsphere is a well-established treatment option in HCCs. Recent data established increasing complete response rates in cases whose dose were planned with personalized volumetric multi-compartment dosimetric approach. In the first insight, centrally located HCCs could be considered only for palliative intent. We put forward that objective treatment responses were similar in these patients compared with peripherally located ones.

## Data Availability

The datasets generated or analyzed during the study are not publicly available due to they relate to a novel radiopharmaceutical but are available from the corresponding author on reasonable request.
